# Identifying unusual performance in Australian and New Zealand intensive care units from 2000 to 2010

**DOI:** 10.1186/1471-2288-14-53

**Published:** 2014-04-22

**Authors:** Patricia J Solomon, Jessica Kasza, John L Moran

**Affiliations:** 1 School of Mathematical Sciences, University of Adelaide, North Terrace, 5005 Adelaide, Australia; 2 Department of Epidemiology and Preventive Medicine, Monash University, 3004 Melbourne, Australia; 3 Department of Intensive Care Medicine, The Queen Elizabeth Hospital, 28 Woodville Road, Woodville, 5011 Adelaide, Australia

**Keywords:** Hierarchical models, Hospital comparisons, Intensive care performance, Multiple comparisons, Risk-adjusted mortality, Seasonal effects, Variance components

## Abstract

**Background:**

The Australian and New Zealand Intensive Care Society (ANZICS) Adult Patient Database (APD) collects voluntary data on patient admissions to Australian and New Zealand intensive care units (ICUs). This paper presents an in-depth statistical analysis of risk-adjusted mortality of ICU admissions from 2000 to 2010 for the purpose of identifying ICUs with unusual performance.

**Methods:**

A cohort of 523,462 patients from 144 ICUs was analysed. For each ICU, the natural logarithm of the standardised mortality ratio (log-SMR) was estimated from a risk-adjusted, three-level hierarchical model. This is the first time a three-level model has been fitted to such a large ICU database anywhere. The analysis was conducted in three stages which included the estimation of a null distribution to describe usual ICU performance. Log-SMRs with appropriate estimates of standard errors are presented in a funnel plot using 5% false discovery rate thresholds. False coverage-statement rate confidence intervals are also presented. The observed numbers of deaths for ICUs identified as unusual are compared to the predicted true worst numbers of deaths under the model for usual ICU performance.

**Results:**

Seven ICUs were identified as performing unusually over the period 2000 to 2010, in particular, demonstrating high risk-adjusted mortality compared to the majority of ICUs. Four of the seven were ICUs in private hospitals. Our three-stage approach to the analysis detected outlying ICUs which were not identified in a conventional (single) risk-adjusted model for mortality using SMRs to compare ICUs. We also observed a significant linear decline in mortality over the decade. Distinct yearly and weekly respiratory seasonal effects were observed across regions of Australia and New Zealand for the first time.

**Conclusions:**

The statistical approach proposed in this paper is intended to be used for the review of observed ICU and hospital mortality. Two important messages from our study are firstly, that comprehensive risk-adjustment is essential in modelling patient mortality for comparing performance, and secondly, that the appropriate statistical analysis is complicated.

## Background

Comparing the performance of intensive care units (ICUs) is important for health care provider accountability and for ensuring public safety. In this paper, we compare ICUs contributing to the Australian and New Zealand Intensive Care Society (ANZICS) Adult Patient Database (APD) from 2000 to 2010. The purpose of the comparison is to identify ICUs with unusual performance as characterised by risk-adjusted in-hospital mortality. Such a characterisation of performance is not without controversy, but we take the pragmatic view that comparisons of mortality will be undertaken, and that it is important the analysis be conducted in a statistically rigorous manner. In-hospital mortality is the only widely available mortality measure for the ANZICS APD, as population mortality databases are maintained by separate State and Territory jurisdictions in Australia and they are not currently linked. In any case, the analysis of 30-day mortality or patient survival is controversial in this context, [1,2].

The ANZICS APD is one of the largest binational databases in the world. In 2010, 124 of the eligible 157 ICUs contributed to the database which currently contains more than one million intensive care patient submissions collected from ICUs in Australia and New Zealand. The ANZICS APD has been collecting data since 1987 on physiological and chronic health status variables at the point of ICU admission and over the subsequent 24 hours, [3]. The specific variables are primarily relevant to the computation of hospital mortality probabilities for existing algorithms such as APACHE II and III, and SAPS II, [4-6]. Mortality outcome is recorded at ICU and hospital discharge.

Ours is the first comprehensive, risk-adjusted analysis of mortality in the ANZICS APD covering such an extended period and provides the most complete picture to date of critical care outcomes in Australia and New Zealand. Patient mortality is modelled using a risk-adjusted three-level hierarchical logistic regression model which clusters patients within years and years within ICUs. Hierarchical models are also known as multilevel models and the three levels of the hierarchy are: between ICUs (level three), between years within ICUs (level two, treating years as independent random effects, which we refer to as ICU-years) and between patients within ICU-years within ICUs (level one). Such models capture the hierarchical nature of the data and the fact that responses within clusters are correlated. The use of hierarchical models for the assessment of health care provider performance has been recommended as best practice [7-9], and is standard for the analysis of hospital outcomes data [10-12]. However, hierarchical models appear much less frequently in the critical care literature and ours is the first application of a three-level hierarchical model to ICU comparisons. Furthermore, our statistical approach using a three-stage analysis includes the estimation of a null distribution to describe ‘usual performance’. Our analysis extends the work of Ohlssen *et al.*[13] from a Bayesian to a classical (frequentist) framework using empirical Bayes models, and from a two-level to a three-level hierarchical model and application to longitudinal data, [14].

We have chosen the standardized mortality ratio (SMR) as the performance indicator on which to base the ICU comparisons. For each ICU, this is the ratio of the observed to expected number of deaths, where a value of one implies that the two numbers are in agreement under the assumptions of the model. The SMR is widely used in applications of provider comparisons and in mortality studies generally, and has the attractive feature of incorporating both the observed and expected numbers of deaths. Typically when hierarchical models are used to characterise mortality, providers are compared using random effects estimators. These estimators provide shrinkage towards the overall mean and can be of substantial benefit when incorporating low-volume providers in the analysis. However, the estimates are also potentially biased, [15]. Our study assumes a minimum annual volume of 150 patient admissions to each ICU in each year, thereby avoiding instability of estimation issues or potential bias due to excessive shrinkage. Bias can also arise when patient characteristics are associated with provider attributes and we expand on this further in the Discussion. Our overall statistical approach avoids problems apparently associated with simplistic (direct and indirect) casemix adjustment methods [16] and mis-interpretation which can arise when comparing hospitals using funnel plots (again under artificially abridged assumptions) with small observed numbers of deaths [17]. Recent statistical work in the social sciences has shown that large numbers are required for valid inference at each level of the hierarchies modelled, [18], a condition our study of ICU performance in the ANZICS APD more than satisfies.

## Methods

### Data

Patient data from 2000 to 2010 were extracted from the ANZICS APD, [3,19]. The initial dataset contained 858,758 admissions from 1,354 ICU-year units from 161 ICUs. Exclusions were patients with: unknown hospital outcome (18,244); ICU length-of-stay (LOS) ≤4 hours (9,607); age <16 years (14,752); coronary artery bypass graft (CABG) (81,166); ICU admissions for the same and separate hospital admissions (123,151); and missing Acute Physiology and Chronic Health Evaluation (APACHE) III score, age, ventilation status, diagnostic category, or ICU source (40,507). Patients with CABG and uncomplicated acute myocardial infarction are not considered in the current dataset, and the exclusion of patients with LOS ≤ 4 hours was an original APACHE III requirement [5]. To ensure stability of estimation, ICU-year units with fewer than 150 complete patient records were excluded; this corresponds to three or fewer admissions per week to an ICU in a year. The final dataset consisted of records for 523,462 patients from 984 ICU-year units from 144 ICUs. Access to the data was granted by the ANZICS Database Management Committee in accordance with standing protocols. The research was exempt from formal University of Adelaide Human Research Ethics Committee approval according to the Australian National Statement on Ethical Conduct in Human Research, 2007 and local hospital (The Queen Elizabeth Hospital, Adelaide) Human Research Ethics Committee approval was waived.

### Statistical methods

#### Performance indicator

The performance indicator is the natural logarithm of the standardised mortality ratio (log-SMR), where the SMR is the ratio of the observed to expected numbers of in-hospital deaths. We prefer the log to the raw SMR, as it provides confidence intervals with better coverage probabilities and is approximately normally distributed, [14,20].

#### Identifying unusual ICUs

##### 

**Stage 1: Risk-adjusted model** The dataset was split randomly into 80% training and 20% test datasets for model building. Owing to the (very long) computing time required to fit three-level models to such a large dataset, the initial model selection was conducted using one-level models and the logistic command in Stata™ (Version 12, 2011, [21]). Continuous (fixed) covariates included APACHE III score, year of admission, and annual ICU volume. The inclusion of Year as a fixed effect enabled adjustment for a (systematic) trend in the response over time. The patient severity score APACHE III is the third revision of the Acute Physiology and Chronic Health Evaluation (APACHE III) score, which is computed using the patient’s worst values during the first 24 hours post-admission to the ICU, [5]. The APACHE III score is the most referenced patient severity of illness score in the clinical literature and is an important predictor of mortality, [22]. It has a range 0-299, and in the original paper, [5], the mean score was 50. A severely-ill patient would have an APACHE III score of between 50-70. It is an important (although not the sole) measure of patient illness, since it combines several physiological and chronic health status variables. Our previous work fitting random intercept and random coefficient logistic regression models to the ANZICS APD demonstrated that APACHE III is the most important predictor of mortality, [14,19]. In the present study, APACHE III was fitted as a non-linear term (in particular, a degree four polynomial) with the inclusion of a random slope term for APACHE III as this significantly improved the goodness of fit. Additional random terms were not included to avoid increasing the complexity of the model and the associated computing time. Age was fitted as a grouped variable with six bins, which enabled better prediction for very elderly patients. Fitting splines or fractional polynomials, [23], did not improve the fit of age or APACHE III. Descriptors of ICU-admission primary-organ-system dysfunction and patient surgical status (*i.e.*, patient diagnostic category) were generated by consolidating the diagnostic categories of the APACHE III algorithm. ICU-level variables locality and hospital level were also included in the model. The discrete explanatory variables were fitted as indicator variables. Increased mortality during the winter months and at weekends has previously been observed in the ANZICS APD [24,25]. This was modelled here by sine and cosine trigonometric terms representing yearly and weekly periods and initially included harmonic terms at six months and 3.5 days, calculated from calendar day of admission, [26,27]. However, the harmonic terms at six months and 3.5 days were not significantly associated with mortality and were dropped from the final model. Interactions between the periodic terms and locality were also included in the model, together with other clinically meaningful two-way interactions. Continuous explanatory variables were centred for model fitting and variables were removed stepwise if the estimated *p*-value was >0.05, excepting the pairs of sine and cosine terms which were always retained together in the model. Annual volume was retained in the final model because it is important, and Year squared was included to allow for a (systematic) nonlinear trend over time. Fitted models were compared using AIC (for nested models only), BIC, area under the ROC curve, and the Hosmer-Lemeshow test statistic (the latter used with caution in this large dataset, [28]). Binned residual plots were used to assess both the covariate modelling and the overall model fit and to help select a final model, [29].

A three-level risk-adjusted hierarchical model based on the best fitting one-level model was fitted to the dataset using Stata™’s xtmelogit command and the Laplace approximation, [21]. Preliminary investigations using two-level hierarchical models and simulations demonstrated that the parameter estimates (and their standard errors) obtained using the Laplace approximation versus seven-point Gaussian quadrature were almost the same. If *Y*_*itj*_ represents the in-hospital mortality outcome (1 for death, 0 otherwise) for patient *j* in year *t* in ICU *i*, and *P*_*itj*_ is the binomial probability of in-hospital mortality for this patient, the log odds of death is given by

(1) $$\text{logit}(P_{\text{itj}})=\beta_{0}+\overset{K}{\underset{k=1}{\sum }}\beta_{k}X_{\text{itjk}}+U_{\text{it}}+U_{i}+U_{i1}\text{APACHE III},$$

where *X*_*itjk*_ contains the observed (fixed-effects) explanatory variables for patient *j*, *U*_*it*_ is the random intercept for year *t* in ICU *i*, *U*_*i*_ is the random intercept for ICU *i*, and *U*_*i*1_ is the random coefficient for APACHE III score. The indices range from *i* = 1,…,144 ICUs, *t* = 1,…,*n*_*i*_ years within ICU *i*, and *j* = 1,…,*n*_*it*_ patients within ICU-year *t*. The level-three (ICU-level, or between ICUs) random intercepts are assumed to be normally distributed with zero mean and variance $\sigma_{I}^{2}$; the APACHE III slopes are also assumed normally distributed with variance $\sigma_{\text{AP}}^{2}$, and there is a component of covariance, *σ*_*I*,*AP*_, assumed at level three. The level-two (ICU-year, or between years within ICUs) random effects are assumed to be independently normally distributed with variance $\sigma_{\text{IY}}^{2}$, independently of the level-three random effects. The level-three random intercepts represent (potentially unknown) differences between ICUs and the random slopes for APACHE III allow the dependence of in-hospital mortality on patient severity to vary between hospitals. The component of covariance accommodates potential dependence between the intercept and APACHE III slope terms within ICUs. The normality assumptions for the random effects were assessed using estimated gradient graphs, [30].

Using approximate cross-validation, we assessed how well the observed data from each ICU were fitted by the final model, [14]. This involved estimating an approximate *p*-value for each ICU from the three-level hierarchical model as follows. To begin, random effects were simulated from the fitted random effects distribution (1) 5,000 times. Given the simulated random effects, the probability of death for patient *j* in ICU-year *t* in ICU *i*, ${\tilde{P}}_{\text{itj}}$, was calculated using the fitted model. Then $Y_{\text{itj}}^{k}$, the corresponding outcome for iteration *k* = 1,…,5,000, was simulated from a Bernoulli distribution with probability of success ${\tilde{P}}_{\text{itj}}$; so $Y_{\text{itj}}^{k}$ is equal to 0 or 1. Then the simulated number of deaths for ICU *i* was calculated as $E_{i}^{k}=\sum_{t=1}^{n_{i}}\sum_{j=1}^{n_{\text{it}}}Y_{\text{itj}}^{k}$. For each ICU, the proportion of times the observed number of deaths, *O*_*i*_, exceeded the simulated number of deaths was calculated as

$$p_{i}-\text{approx}=\frac{1}{5000}\overset{5000}{\underset{k=1}{\sum }}I_{E_{i}^{k}<O_{i}},$$

where *I* is the indicator function. This gave an approximate *p*-value for each ICU under the nominal null hypothesis that the SMR is equal to one. Under this null hypothesis, we would expect the simulated number of deaths to exceed the observed number in approximately half of the simulations, [14]. Thus *p*-approx measures how well the estimated model predicts the observed number of deaths for each ICU. We chose a nominal 20% significance level for this first stage of screening for potential outliers. When *p*-approx < 0.1, an ICU was assessed to be potentially over-performing (*i.e.*, has low mortality), and when *p*-approx > 0.9, a site was potentially under-performing (*i.e.*, has high mortality). It may be helpful to plot a histogram of the *p*-values, or transformed *p*-values, to detect the presence of outliers. If the variability amongst the providers is very small with no obvious outliers, one might decide on a much lower nominal level of significance such as 5% or to proceed with a different analysis for comparison, or no analysis.

##### 

**Stage 2: A null model** The Stage 2 model was estimated by excluding the potentially unusual ICUs identified in Stage 1, then re-fitting the final model. This provided a null ‘reference’ distribution for describing usual ICU performance. Log-SMRs and their variances were again estimated for each ICU, according to the methods described in [14] and Additional file 1. The estimation of the variances of the log-SMRs is somewhat technical, but an outline of the calculations to obtain the approximate variance of the log-SMR for ICU *i* in year *t* is given in Additional file 1. The uncertainty in estimating the expected number of deaths for each ICU is therefore accounted for in our analysis, whereas this is usually treated as given. Treating the estimated expected number of deaths as a constant in the calculations under-estimates the true variance of the log-SMRs, so our analysis offers an advantage over what is usually done. Note that the potentially unusual ICUs were modelled without random effects, so for each unusual ICU, a usual ICU was randomly selected and the random effects predictions from that ICU used to calculate the expected number of deaths for the potentially unusual ICU. Extensive sensitivity analyses demonstrated that randomly selecting the random effects from the ‘usual’ distribution in this way gave the same results as stratifying on ICU level, for example.

##### 

**Stage 3: Unusual ICUs** The funnel plot was constructed as described previously, [14]. ICUs with log-SMRs lying outside the funnel were identified as performing unusually, with either higher or lower mortality than usual. All ICUs have been randomly allocated a random identity number which is shown for those lying outside the thresholds. Confidence intervals controlling the false coverage-statement rate (FCR) at 5% were also constructed for the ICUs identified as unusual, [31]. The FCR is the expected proportion of false discovery rate (FDR) selected [32] confidence intervals which do not cover their true parameter values. FCR is a property of the set of confidence intervals not covering zero and does not involve confidence intervals for the non-selected parameters. However, all confidence intervals may be plotted together by applying visual impact to distinguish the two sets of intervals (selected and non-selected) and we use bold lines to distinguish the FDR-selected intervals. The remaining intervals have FCR coverage of at most 0.05 for all parameters because the FCR offers marginal coverage of at least 0.95. We further evaluated the performance of the outlying ICUs by posing the question: is the worst ICU worse than expected, given it has arisen from the null (usual) predictive distribution, [13]? This question is answered by simulating the distribution of the predicted true worst number of deaths and comparing it to the observed worst number of deaths.

## Results

### Data

The mean(sd) for age was 60.6(18.8) years and for APACHE III score, 51.5(28.6); 57.0*%* of patients were male and 12.8*%* of patients died in hospital. Patient characteristics for the entire dataset are given in Table 1. The number of patient records increased each year from 20,888 in 2000 to 74,342 in 2010, and the number of contributing ICUs increased from 44 to 122 over the same period. Hospital mortality declined steadily each year, from 17.3*%* in 2000 to 10.5*%* in 2010. New South Wales (NSW) had the largest number of ICUs and patients, overall and in each year. Patient characteristics by year are given in Table 2, and Table 3 sets out the ICU characteristics by year and geographical locality.

**Table 1:** Characteristics of the 523,462 ANZICS APD patients analysed

Age (years)	60.6 (18.1)	
APACHE III score	51.5 (28.6)	
ICU mortality (%)	8.1	
Hospital mortality (%)	12.7	
	n (%)	mortality %
**Ventilation**		
Not ventilated	314,987 (60.2)	7.4
Ventilated	208,475 (39.8)	20.6
**Gender**		
Male	298,503 (57.0)	12.2
Female	224,959 (43.0)	13.0
**ICU source**		
No transfer	478,130 (91.3)	12.1
Hospital transfer	45,332 (8.7)	18.7
**ICU hospital level**		
Rural	71,828 (13.7)	11.5
Metropolitan	98,590 (18.8)	14.7
Tertiary	232,273 (44.4)	15.9
Private	120,771 (23.1)	5.6
**ICU location**		
Northern Territory	8,965 (1.7)	12.5
New South Wales	153,362 (29.3)	13.2
Australian Capital Territory	15,815 (3.0)	10.1
South Australia	37,052 (7.1)	19.3
Victoria	137,876 (26.3)	12.8
Western Australia	5,493 (1.1)	11.7
New Zealand	27,835 (5.3)	15.6
Queensland	126,453 (24.2)	9.5
Tasmania	10,611 (2.0)	14.7

Age and APACHE III score are given as mean (sd).

**Table 2:** Characteristics of ANZICS APD study patients by year, 2000-2010

**Hosp. admit year**	***n* (%)**	**Hosp. mort. (%)**	**ICU mort. (%)**	**APIII mean (sd)**	**Age mean (sd)**	**Vent. (%)**	**Transfer (%)**
2000	20,888 (4.0)	17.3	11.1	53.7 (30.9)	58.9 (19.3)	48.1	8.9
2001	26,353 (5.0)	15.8	10.1	52.6 (30.3)	59.6 (19.2)	44.0	9.6
2002	32,380 (6.2)	15.3	9.9	51.7 (29.7)	60.0 (18.9)	42.6	9.4
2003	37,082 (7.1)	14.4	9.2	51.5 (29.0)	60.4 (18.8)	41.0	9.1
2004	43,132 (8.2)	13.6	8.5	51.5 (28.4)	60.7 (18.6)	40.3	8.8
2005	49,093 (9.4)	12.9	8.2	50.9 (28.4)	60.6 (18.6)	40.1	8.8
2006	54,323 (10.4)	12.1	7.8	51.0 (28.2)	61.1 (18.8)	38.5	8.4
2007	57,187 (10.9)	12.0	7.8	51.0 (28.4)	61.0 (18.7)	37.6	8.5
2008	61,667 (11.8)	11.7	7.5	51.8 (28.7)	60.8 (18.8)	39.3	8.4
2009	67,015 (12.8)	11.3	7.3	51.8 (28.4)	60.8 (18.8)	39.3	8.4
2010	74,342 (14.2)	10.5	6.8	50.8 (27.6)	61.1 (18.8)	37.5	8.3

**Table 3:** ICU characteristics by year and geographical locality of Australia, or New Zealand

**Hosp. admit year**	**NT**	**NSW**	**ACT**	**SA**	**VIC**	**WA**	**NZ**	**QLD**	**TAS**	**Total ICUs**
2000	543 (2)	7,454 (16)	754 (1)	1,784 (3)	5,622 (11)	0	0	4,367 (10)	364 (1)	44
2001	386 (1)	8,572 (18)	1,019 (2)	1,814 (3)	7,186 (16)	0	799 (2)	5,726 (12)	851 (2)	56
2002	586 (2)	9,390 (20)	1,412 (3)	2,040 (4)	9,075 (19)	0	1,018 (2)	7,874 (17)	985 (3)	70
2003	884 (2)	10,149 (21)	1,479 (3)	1,623 (3)	11,195 (19)	0	1,565 (4)	9,123 (20)	1,064(3)	75
2004	1,119 (2)	11,331 (24)	1,460 (3)	2,328 (5)	12,912 (23)	162 (1)	1,987 (5)	11,081 (22)	752 (2)	87
2005	997 (2)	12,555 (26)	1,650 (3)	2,937 (6)	13,638 (24)	530 (2)	3,214 (6)	12,452 (25)	1,120 (3)	97
2006	954 (2)	14,917 (29)	1,723 (3)	4,369 (6)	14,746 (25)	511 (2)	3,050 (7)	12,903 (25)	1,150 (3)	102
2007	945 (2)	16,908 (33)	1,861 (3)	4,957 (6)	14,665 (24)	295 (1)	3,554 (8)	13,057 (25)	945 (2)	104
2008	819 (2)	17,728 (33)	1,272 (2)	4,989 (7)	15,554 (27)	1,101(1)	3,687 (8)	15,420 (29)	1,097 (2)	111
2009	808 (2)	20,551 (36)	1,536 (2)	5,253 (6)	15,607 (27)	1,423 (2)	3,702 (7)	16,895 (31)	1,240 (3)	116
2010	924 (2)	23,807 (38)	1,649 (2)	4,958 (6)	17,676 (29)	1,471 (2)	5,259 (10)	17,555 (31)	1,043 (2)	122
Total	8,965 (2)	153,362 (46)	15,815 (3)	37,052 (9)	137,876 (34)	5,493 (3)	27,835 (11)	126,453 (33)	10,611(3)	144

The total number of patients in the dataset for each year/locality pair is given, followed by the number of ICUs for each pair in brackets.

### Identifying unusual ICUs

#### 

**Figure 1:**
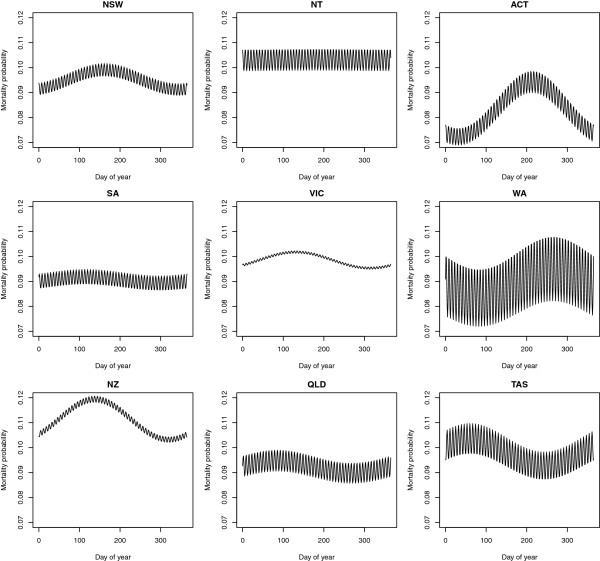
**Yearly and weekly respiratory seasonal effects across Australia and New Zealand in 2010.** Estimated annual cycle from 1 January (day 1) to 31 December (day 365) and weekly cycle Saturday to Friday, for Australian States and Territories and New Zealand. The seasonal effects are for 2010 respiratory patients, conditional on the estimated Stage 1 model. Queensland (QLD) and the Australian Capital Territory (ACT) have significantly earlier (March) and later (August) peak mortalities compared to New South Wales (NSW), which peaks in June. QLD and Tasmania (TAS) have weekly peak mortalities associated with admissions on Mondays and Wednesdays respectively, compared to NSW which has weekly peak mortality associated with Saturday admissions. No other cycles differ significantly from NSW. **Legend**: NT, Northern Territory; SA, South Australia; VIC, Victoria; WA, Western Australia; NZ, New Zealand.

**Stage 1: Risk-adjusted model** The final model took 5.7 days to converge on a 6-core computer with 3.30 GHz CPU. The ROC AUC was equal to 0.90 and the H-L statistic was equal to 0.003 (*p* > 0.40). There were 150 fixed effects parameters estimated as log odds which are set out in Additional file 2. Estimates of the four components of variance and covariance (with their standard errors) are given in Table 4. There were no model convergence issues. As would be expected in such a large dataset, the normality assumptions were valid according to the estimated gradient graphs shown in Additional file 3. A significant linear decline in mortality over the decade was observed (the log odds of death for Year decreased by -0.06 per year, 95% CI (-0.078,-0.036)). Indeed, excepting Annual volume and Year squared, all the explanatory variables and interactions in Additional file 2 are statistically significantly associated with the log odds of death. Note that the table in Additional file 2 includes all the explanatory variables fitted in the final model. ICUs in private sector hospitals are observed to have a significantly lower log odds of death compared to tertiary (usually large, teaching) hospitals. The seasonal terms are significantly associated with geographical locality, and Figure 1 shows risk-adjusted yearly and weekly seasonal effects for Australian and New Zealand respiratory diseases in 2010. Interactions were estimated relative to the largest state, New South Wales (NSW), continuous covariates were assumed at their average values, and categorical variables have been taken at baseline. Figure 1 shows that ICU mortality in the Australian Capital Territory (ACT) peaked significantly later in the year than NSW (August compared to June) whereas Queensland (QLD) peaked significantly earlier in March. The Northern Territory (NT) had no apparent annual seasonal effect. Note that some of the estimated standard errors (not shown) are large. There were also differences in weekly peak mortalities across jurisdictions. NSW, ACT and the NT peaked on Saturday admissions, whereas QLD had a statistically significant peak on Mondays, and Tasmania (TAS) on Wednesdays. New Zealand had peaks in May and on Wednesdays but the differences are not statistically significant. Note that the cycles correspond to day of admission. We also note that this risk-adjusted approach to modelling seasonality is an advance over the methods employed in [33].

**Table 4:** Components of variance and covariance from the Stage 1 and Stage 2 models

	**Stage 1**	**Stage 2**
ICU-level intercept	0.077640	0.034902
	(0.012262)	(0.006669)
APIII slope	0.027744	0.027451
	(0.004727)	(0.005093)
Cov(ICU-level intercept, APIII)	-0.023946	-0.021549
	(0.006197)	(0.004955)
ICU-year-level intercept	0.019852	0.020039
	(0.002474)	(0.002650)

Estimated standard errors are given in brackets.

**Figure 2:**
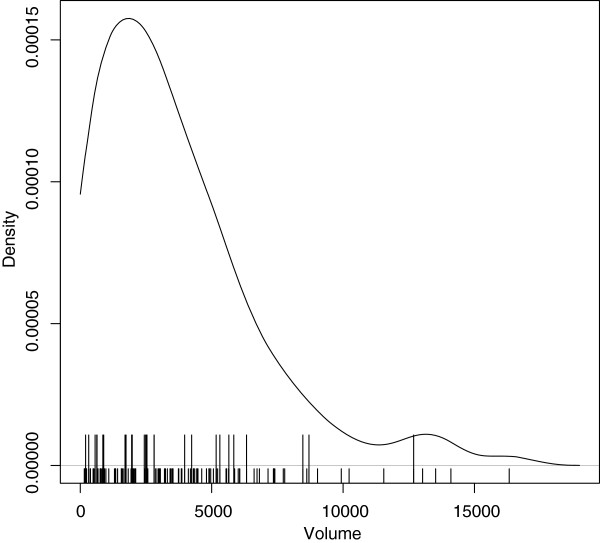
**Kernel density plot of total ICU volume over 2000-2010.** Large tick-marks indicate volumes of ICUs deemed to be potentially unusual at Stage 1 of the analysis.

*Identifying potentially unusual ICU performance*: Over the period of observation, 27 ICUs were identified as potentially unusual. There were 14 over-performing ICUs with *p* - approx < 0.1 and 13 under-performing ICUs with *p* - approx > 0.9; the results are set out in Table 5. More than half (16) of these ICUs were in private hospitals; note that the site numbers are random, not APD site IDs. Figure 2 shows a kernel density plot of total ICU volume over 2000-2010. The potentially unusual ICUs are indicated by large tick marks and the plot shows that their volumes are reasonably evenly distributed over the entire volume range, and therefore not confounded with performance.

**Table 5:** Hospital level and locality for the 27 ICUs identified as unusual at Stage 1

**Random ID**	**Hospital Level**	**Locality**	**Years**	***p-approx***
100	Private	New South Wales	2006-10	0.002
57	Private	New South Wales	2007-10	0.008
116	Private	New South Wales	2005-08	0.010
79	Metropolitan	Queensland	2004-10	0.015
131	Metropolitan	New South Wales	2000-02	0.017
18	Private	Queensland	2003-06, 08-10	0.023
72	Rural	Victoria	2008-10	0.025
120	Private	Australian Capital Territory	2001-07	0.027
108	Private	New South Wales	2000-10	0.030
49	Metropolitan	Victoria	2001, 04, 08-10	0.038
66	Tertiary	South Australia	2000-10	0.045
24	Private	Queensland	2001-10	0.058
112	Rural	Victoria	2002-10	0.065
19	Private	New South Wales	2002-10	0.080
54	Tertiary	South Australia	2000-10	0.915
2	Tertiary	New South Wales	2008-10	0.917
89	Private	South Australia	2005-06, 08-10	0.928
129	Private	New South Wales	2010	0.938
95	Private	Victoria	2001-06, 09	0.942
134	Metropolitan	Victoria	2003-08	0.942
81	Private	Victoria	2009-10	0.950
104	Metropolitan	Queensland	2000-10	0.953
44	Private	Queensland	2002-10	0.953
16	Private	Queensland	2001-10	0.972
93	Private	Queensland	2009-10	0.978
91	Metropolitan	New South Wales	2008-10	0.987
140	Private	Victoria	2000-02	0.997

14 ICUs with *p* - approx less than 0.1 are over-performers (low mortality) and 13 ICUs with *p* - approx greater than 0.9 are under-performers (high mortality). The Years column gives the years the ICU contributed to the dataset.

#### 

**Stage 2: A null model** The concern is that the potentially unusual ICUs are inflating the estimates of the random effects distribution at Stage 1. The 27 potentially unusual ICUs identified in Stage 1 were therefore excluded from the Stage 2 analysis, which resulted in *n*=430,049 patients in 816 ICU-years from 117 ICUs. The three-level hierarchical logistic regression model was then re-fitted to the reduced dataset. This resulted in good agreement between the fixed effects parameter estimates from both Stages as shown in Additional file 2. However, the variance component estimate corresponding to the Stage 2 ICU-level random intercept was reduced by more than half from Stage 1, as shown in Table 4. The ICU-year intercepts variance component estimates were little changed between the two stages, and similarly for the between-ICU APACHE III slope variance components estimates. The estimated component of covariance between the ICU-level intercept and slope was slightly reduced at Stage 2, and remained rather small (-0.022, Table 4). This indicates that the ICU-level random intercepts are representing unexplained differences between ICUs, whereas differences between-years within-ICUs are similar across sites. The approach used here to attenuate the effects of the potentially unusual ICUs differs from that in [14] where all ICUs contributed to the estimation of the fixed effects component of the model, and has been taken primarily for computational reasons. As discussed below, the results here are in excellent agreement with those obtained previously.

#### 

**Figure 3:**
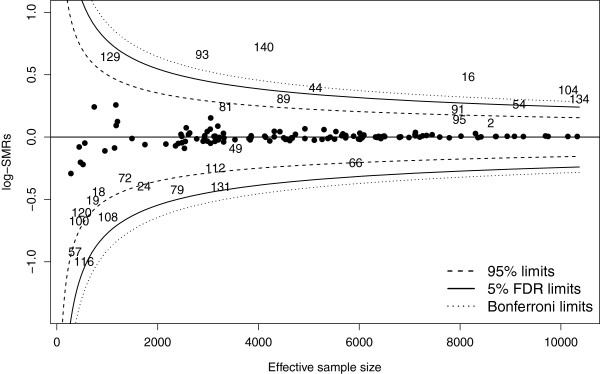
**Funnel plot of log-SMRs versus effective sample size for each ICU from Stage 2 of the analysis.** The funnels correspond to 95% classical limits (dashed lines) not adjusted for multiple testing, the Bonferroni limits controlling the FWER at 5% (dotted lines), and limits controlling the FDR at 5% (solid lines). Potentially unusual ICUs are marked with their random identifying numbers. The effective sample size is the estimated variance of the log-SMR as a fraction of the total variance. **Legend**: SMR: standardised mortality ratio. FDR: false discovery rate. FWER: family-wise error rate.

**Stage 3: Unusual ICUs** Figure 3 displays the funnel plot of the estimated log-SMR versus its estimated precision for each ICU. Controlling the FDR at 5%, seven ICUs (140,16,93,104,44,134,54) were identified as having unusually poor performance over the study period. No ICUs were identified as having unusually good performance at the 5% FDR level of significance. Using the classical limits (not adjusted for multiple comparisons), 15 ICUs were identified as having unusually good or poor performance. ICUs 89 and 129 were also identified as performing poorly, whereas ICUs 57,66,79,108,116 and 131 were identified as performing well, with low mortality. The 5% Bonferroni thresholds are shown on the funnel plot for comparison, to demonstrate that controlling the family-wise error rate makes it harder to identify unusual performance in this setting.

**Figure 4:**
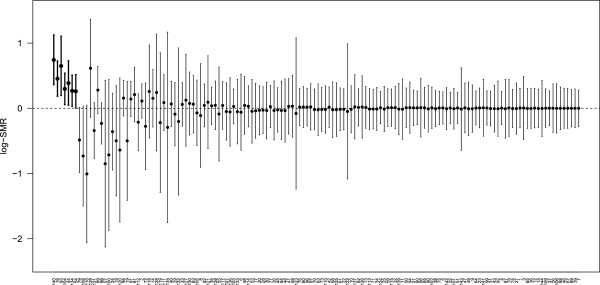
**Five percent false coverage-statement rate confidence interval estimates for all 144 ICUs.** The seven 5% FDR-selected ICUs identified as unusual are highlighted in bold on the left; these ICUs have confidence intervals with the correct FCR rate of 0.05. The remainder have FCR coverage of at most 0.05 for all parameters because the FCR offers marginal coverage of at least 0.95. The ICUs are ordered from those with the smallest to the largest *p*-values. **Legend**: FDR: false discovery rate. FCR: false coverage-statement rate.

Figure 4 presents an alternative, confidence interval display of the results using the FCR. The seven FDR-selected ICUs are distinguished visually by vertical bold lines on the left-hand-side of the plot; this is to emphasise that the selected parameters have the correct FCR coverage of 0.05. The FCR is a property of the set of confidence intervals not covering zero. The remaining confidence intervals have FCR coverage of at most 0.05 for all parameters because the FCR offers marginal coverage of at least 0.95.

**Figure 5:**
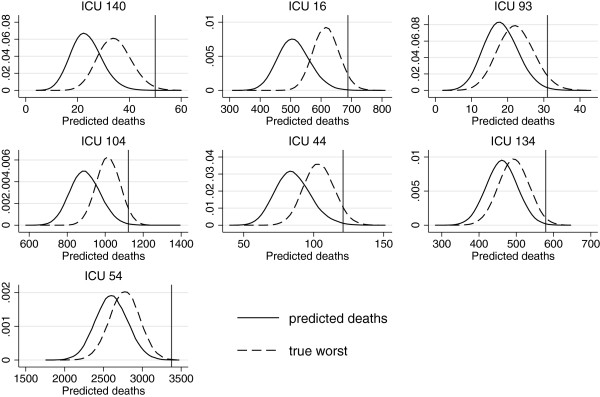
**Predicted distributions of the ‘true worst’ numbers of deaths for each of the seven unusual ICUs.** Each subplot shows the simulated probability density functions for the predicted number of deaths and predicted ‘true worst’ number of deaths for the seven ICUs identified as unusual. Each simulation is based on 50,000 replications. The observed number of deaths is indicated by the solid vertical line in each case. The plots are presented in order of the true worst (ICU 140), second true worst (ICU 16), and so on, up to the seventh true worst (ICU 54).

**Figure 6:**
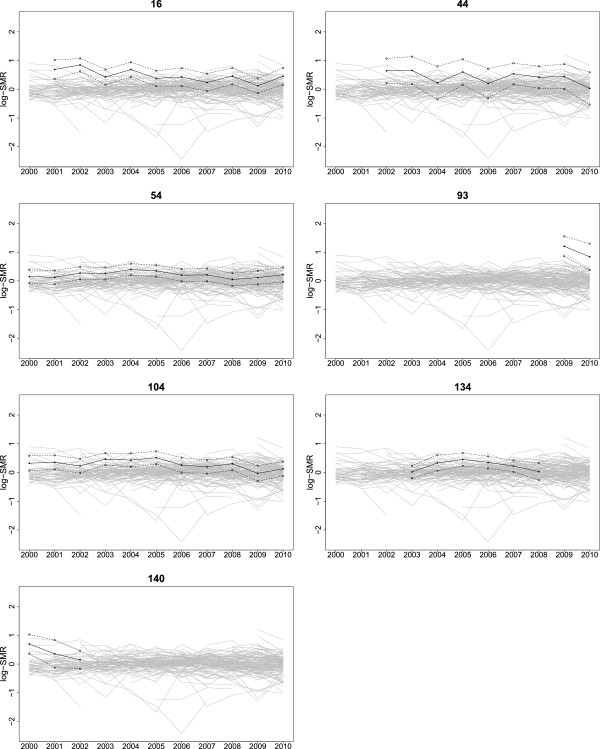
**Yearly estimated log-SMRs plotted over time for the seven unusual ICUs.** In each case, the yearly log-SMRs are shown by bullets joined by solid lines with ± (plus and minus) two standard errors marked by open circles joined by dashed lines. The ICUs are presented in random-identity number order.

Figure 5 shows the results of simulating from the null predictive and the true worst null predictive probability density functions, for the seven unusually performing ICUs. The plots demonstrate that the observed numbers of deaths for these ICUs do not sit within the null predictive distributions for usual performance. Figure 6 displays time-plots for the yearly estimated log-SMRs with estimated 95% confidence intervals for the seven unusual ICUs identified.

## Discussion

Seven ICUs were identified with unusually high mortality by our analysis. ICUs 16,44 and 93 have been previously identified in an analysis of data from 2009-2010, [14], and the present longitudinal study confirms those findings. ICU 81 was also identified with unusually high mortality in [14], and was labelled as potentially unusual at Stage 1. We note that ICUs 16,44 and 93 are in private hospitals in Queensland. A total of 23% of patient admissions were to private sector ICUs which cover most areas of care (medical and surgical) and includes end-of-life care. A recent study comparing conventional risk-adjusted fixed and random effects models analysing the 2009-2010 data was unable to detect any outliers (Moran and Solomon, preprint submitted for publication, 2014).

The ‘Swiss cheese’ nature of the ANZICS APD is apparent from Figure 6, which shows that several ICUs contribute data in some years only. This effect is partly compounded by the minimum 150 patient-volume requirement, and we discuss below issues of missing data and data quality with regard to the particular ICUs identified as unusual. We note that it is by no means assured that the 144 ICUs analysed in this study are representative of the entire adult ICU experience in Australia and New Zealand, and it is likely that the ability to sustain the effort of continuous (and complete) contribution is concentrated in larger, well-resourced ICUs. However, smaller peripheral units are encouraged to participate in the database, and future longitudinal analyses using an instrumental variables approach to participation will help address this issue. Generally, problems with missing data in large observational databases have received a great deal of attention in the literature, and multiple imputation (MI) is the primary technique for handling missing data. However, little of this literature is directed towards MI in the context of hierarchical models, and by way of a caution, a recent simulation study by Twisk *et al.* showed that MI applied to mixed models may be misleading, [34]. Given these observations, the complete case-record analysis we have undertaken is a reasonable way to proceed as a first analysis.

### ICUs with unusual casemix, and data quality

ICU 140 had a very low proportion of ventilated patients compared to comparable ICUs during the three years it contributed data to the ANZICS APD (3.2*%* versus 32.4*%*, *p*-value < 0.0001), and the lowest proportion of ventilated patients of all ICUs in 2000 and 2001. Given ventilation is associated with increased mortality risk, the large log-SMR is associated with the small proportion of ventilated patients. This (historical) outcome could be related to data quality: since ventilation is only inferred from documentation of ventilation at the time an arterial blood gas is taken, there may have been ventilated patients with no blood gas measurement, but we do not know. ICU 54 had the highest proportion of deaths in each year in South Australia. This ICU had a significantly higher proportion of non-surgical patients compared to the other two South Australian tertiary ICUs (72.5*%* versus 51.1*%*, *p*-value < 0.0001) and a higher proportion of deaths amongst non-surgical patients (30.0*%* versus 22.1*%*, *p*-value < 0.0001). However, ICU 54 also had a high proportion of patients with missing APACHE III scores during 2004-2006, and the log-SMRs in 2004 and 2005 were high compared to later years, as shown in Figure 6. ICU 134 also had a high proportion of patients with missing APACHE III scores. It is possible that poor record keeping and high mortality are common causes, for example, of poor ‘process of care’. No association between mortality and important covariates, including APACHE III, could be established at these sites however. Of the remaining ICUs identified with poor performance in our analysis, none had identifiable anomalous casemix, missing data, or other data quality issues. ANZICS CORE analyses outcomes data from ICUs contributing to the APD in a given year using a simple predictive mortality model, based exclusively on the APACHE III algorithm. ICUs identified as outliers by this process are normally followed up according to the Outlier Management Policy [35].

### Variance components

The variance component estimates demonstrate that the staged modelling approach has appropriately accommodated the effects of outliers. The reduction in the Stage 2 ICU-level variance component indicates that the presence of potentially unusual ICUs is inflating the variance component at Stage 1. This component of variance represents differences between ICUs, and the fact that we cannot explain the high mortality for all of the identified ICUs by unusual casemix or other known factors, indicates that there are unexplained differences in mortality between ICUs. At Stage 1, the estimated component of correlation is -0.52. The direct interpretation of this modest correlation is that lower mortality ICU-intercepts are associated with higher APACHE III slopes. This observation is reflecting the fact that the overall average APACHE III score in this dataset increased over the decade at the same time as mortality decreased; a correlation of 0.8 was observed in a normal model of APACHE III on year. Note too that the fitted model, which was estimated using xtmelogit in Stata, allowed for an unstructured covariance matrix and would have detected any important correlations in the random effects distribution. We also note that random effects models are often self-consciously deployed in the literature, and variance components typically treated as nuisance parameters, which they are not here.

### Seasonal effects

Using our Stage 1 mixed model, we have demonstrated for the first time yearly and weekly seasonal effects across Australian jurisdictions. Since it was not the primary purpose of the present paper to study seasonal effects, we have restricted our attention to a comparison conditional on the model baseline variables and baseline patient diagnostic category (*i.e.,* respiratory disease) which is of interest in its own right. Note that the mortality cycles estimated refer to the day of admission, so for example, NSW, the ACT, the NT, VIC, SA and WA all have higher mortality associated with weekend admissions. Tasmania is the only state which has a statistically significant different peak on Wednesday but we do not have an explanation for this yet. New Zealand also has a weekly Wednesday admissions peak, but this was not statistically significantly different from NSW. Interestingly, New Zealand is geographically most similar in climate to Tasmania. Not surprisingly, peak mortalities also tend to occur in the southern hemisphere winter months, which are June, July and August in south-eastern Australia. The tropical Northern Territory has little or no apparent annual seasonal respiratory cycle, and Queensland has a much earlier annual peak mortality. Queensland is a large geographically-diverse state, being tropical in the north and temperate (similar to NSW) in the south. The ACT on the other hand, is located inland, and elevated. It has an annual peak mortality which is later in the year (in August) than anywhere else in Australia.

## Conclusions

The ICUs identified with unusual performance may merit consideration in any future analysis, albeit the observation period studied here is historical. The distinct seasonal mortality patterns identified across regions in Australia undoubtedly warrant further study, from both policy and planning viewpoints. The statistical methods proposed are intended for reviewing and monitoring the performance of ICUs contributing to the ANZICS APD but are appropriate for application to comparable mortality databases. Two key messages from our analysis are firstly, that comprehensive risk-adjustment for patient casemix and factors such as hospital level and locality is essential, and secondly, that the appropriate statistical analysis is complicated.

## Competing interests

The authors declare that they have no competing interests.

## Authors’ contributions

PJS and JLM conceived and designed the study. All authors conducted statistical analysis of the data, with JK performing the substantive component with PJS. All authors were involved in drafting the initial manuscript and PJS and JLM prepared the final version. JK and PJS developed the approximate standard error of the log SMR. All authors read and approved the final manuscript.

## Pre-publication history

The pre-publication history for this paper can be accessed here:

http://www.biomedcentral.com/1471-2288/14/53/prepub

## Supplementary Material

Additional file 1 **Variance of the log-SMR.** The file SE-logSMR.pdf outlines the calculations required to obtain an expression for the approximate variance of the log-SMR for ICU *i* in year *t*. The approximate standard error of the log-SMR is obtained by taking the square root of the variance and is used in the time-plots in Figure 6. Click here for file

Additional file 2 **Table of Stage 1 and Stage 2 model parameter estimates.** Stages 1 and 2.pdf presents all fixed effects parameter estimates (as log odds) from the three-level hierarchical logistic regression models fitted in Stages 1 and 2 of the analysis. The interpretation for the categorical variables is the increase in log-odds of the in-hospital mortality relative to the baseline level (given in brackets). The *p*-value in each case gives the two-sided probability of observing the estimate, or one that is more extreme, under the null hypothesis that the log odds ratio equals zero. Corresponding estimated 95% confidence intervals are also given. Click here for file

Additional file 3 **Estimated gradient function plots.** GradFct.pdf displays the estimated gradient functions for the random effects from the Stage 1 hierarchical model, with point wise confidence intervals in grey, [30]. These plots assess the assumptions of normality made in the model. Normality is assumed to be reasonable if the estimated function (solid line in each case) is close to one (horizontal dotted line) within the limits of the observed data as represented by the vertical dashed lines in each sub-plot. If the estimated gradient function is significantly different from one, the assumption of normality is not valid. The subplots shown in order are for the level-three intercepts, the level-three APACHE III random slopes, and the ICU-year intercepts. As would be expected for such a large dataset as the ANZICS APD, the normality assumptions are satisfied here. Note that a simplifying assumption has been made for the plots presented, in particular, that the random intercepts and random slopes are independent at level-three (ICU-level) of the model. Click here for file

## References

[B1] Schoenfeld D Survival methods, including those using competing risk analysis, are not appropriate for intensive care unit outcome studies Crit Care 2006 10 103 16420653 10.1186/cc3949 PMC1550820

[B2] Freemantle N Richardson M Wood J Ray D Khosla S Shahian D Roche W Stephens I Keogh B Pagano D Weekend hospitalization and additional risk of death: an analysis of inpatient data J R Soc Med 2012 105 74 84 10.1258/jrsm.2012.120009 22307037 PMC3284293

[B3] Stow PJ Hart GK Higlett T George C Herkes R McWilliam D Bellomo R Development and implementation of a high-quality clinical database: the Australian and New Zealand Intensive Care Society Adult Patient Database J Crit Care 2006 21 133 41 10.1016/j.jcrc.2005.11.010 16769456

[B4] Knaus WA Draper EA Wagner DP Zimmerman JE APACHE II: a severity of disease classification system Crit Care Med 1985 13 818 29 10.1097/00003246-198510000-00009 3928249

[B5] Knaus WA Wagner DP Draper EA Zimmerman JE Bergner M Bastos PG Sirio CA Murphy DJ Lotring T Damiano A The APACHE III prognostic system. Risk prediction of hospital mortality for critically ill hospitalized adults Chest 1991 100 1619 36 10.1378/chest.100.6.1619 1959406

[B6] Le Gall JR Lemeshow S Saulnier F A new simplified acute physiology score (SAPS II) based on a European/North American multicenter study JAMA 1993 270 2957 63 10.1001/jama.1993.03510240069035 8254858

[B7] Ash AS Feinberg SE Louis TA Norman SL Stukel TA Utts J Statistical Issues in Assessing Hospital Performance Commissioned by the Committee of Presidents of Statistical Statistical Societies for the Centers for Medicare and Medicaid Services (CMS), January 27, 2012. [ http://www.cms.gov/Medicare/Quality-Initiatives-Patient-Assessment-Instruments/HospitalQualityInits/Downloads/Statistical-Issues-in-Assessing-Hospital-Performance.pdf]

[B8] Gallagher MP Krumholz HM Public reporting of hospital outcomes: a challenging road ahead MJA 2011 194 658 60 21692729 10.5694/j.1326-5377.2011.tb03156.x

[B9] Goldstein H Spiegelhalter DJ League tables and their limitations: statistical issues in comparisons of institutional performance JRSS A 1996 159 285 443

[B10] DeLong ER Peterson ED DeLong DM Muhlbaier LH Hackett S Mark DB Comparing risk-adjustment methods for provider profiling Statist Med 1997 16 2645 64 10.1002/(sici)1097-0258(19971215)16:23<2645::aid-sim696>3.0.co;2-d 9421867

[B11] Jones HE Spiegelhalter DJ The identification of ‘unusual’ health-care providers from a hierarchical model Am Stat 2011 65 154 63 10.1198/tast.2011.10190

[B12] Normand S-LT Shahian DM Statistical and clinical aspects of hospital outcomes profiling Stat Sci 2007 22 206 26 10.1214/088342307000000096

[B13] Ohlssen DI Sharples L Spiegelhalter DJ A hierarchical modelling framework for identifying unusual performance in health care providers JRSS A 2007 170 265 90

[B14] Kasza J Moran JL Solomon PJ Evaluating the performance of Australian and New Zealand intensive care units in 2009 and 2010 Statist Med 2013 13 3720 36 10.1002/sim.5779 23526209

[B15] Kalbfleisch JD Wolfe R On monitoring outcomes of medical providers Stat Bio 2013 5 286 302 10.1007/s12561-013-9093-x

[B16] Pouw ME Peelen LM Lingsma HF Pieter D Steyerberg E Kalkman CJ Moons KGM Hospital standardized mortality ratio: consequences of adjusting hospital mortality with indirect standardization PLOS One 2013 8 4 e59160 doi:10.1371/journal.pone.0059160 10.1371/journal.pone.0059160 23593133 PMC3621877

[B17] Seaton SE Barker L Lingsm HF Steyerberg EW Manktelow BN What is the probability of detecting poorly performing hospitals using funnel plots? BMJ Qual Saf 2013 doi:10.1136/bmjqs-2012-001689 10.1136/bmjqs-2012-001689 23832924

[B18] Bryan ML Jenkins SP Regression analysis of country effects using multilevel data: a cautionary tale ISER Working Paper Series 2013-14 Colchester, University of Essex. [ https://www.iser.essex.ac.uk/publications/workingpapers/iser/2013-14]

[B19] Moran JL Bristow P Solomon PJ George C Hart G Mortality and length-of-stay outcomes, 1993-2003 in the binational Australian and New Zealand intensive care Adult Patient Database Crit Care Med 2008 36 46 61 10.1097/01.CCM.0000295313.08084.58 18090383

[B20] Hosmer DW Lemeshow S Confidence interval estimates of an index of quality performance based on logistic regression models Statist Med 1995 14 2161 72 10.1002/sim.4780141909 8552894

[B21] StataCorp Stata™: Release 12 2011 College Station, TX, USA: StataCorp LP, Statistical Software

[B22] Iezzoni L The risks of risk-adjustment JAMA 1997 278 1600 7 10.1001/jama.1997.03550190064046 9370507

[B23] Sauerbrei W Royston P Binder H Selection of important variables and determination of functional form for continuous predictors in multivariable model building Stat Med 2007 26 5512 8 10.1002/sim.3148 18058845

[B24] Moran JL Solomon PJ Conventional and advanced time series estimation: application to the Australian and New Zealand Intensive Care Society Adult Patient Database 1993-2006 JECP 2011 17 45 60 10.1111/j.1365-2753.2010.01368.x 20807296

[B25] Bhonagiri D Pilcher DV Bailey MJ Increased mortality associated with after-hours and weekend admission to the intensive care unit: a retrospective analysis MJA 2011 194 287 92 21426282 10.5694/j.1326-5377.2011.tb02976.x

[B26] Diggle PJ Time Series: a Biostatistical Introduction 1990 Oxford: Oxford University Press

[B27] Stolwijk AM Straatman H Zielhuis GA Studying seasonality by using sine and cosine functions in regression analysis J Epid Comm H 1999 53 235 38 10.1136/jech.53.4.235 PMC1756865 10396550

[B28] Paul P Pennell ML Lemeshow S Standardizing the power of the Hosmer-Lemeshow goodness of fit test in large data sets Stat Med 2013 32 67 80 10.1002/sim.5525 22833304

[B29] Gelman A Hill J Data Analysis Using Regression and Multilevel/hierarchical Models 2007 Cambridge: Cambridge University Press

[B30] Verbeke G Molenberghs G The gradient function as an exploratory goodness-of-fit assessment of the random-effects distribution in mixed models Biostat 2013 14 477 90 10.1093/biostatistics/kxs059 23376427

[B31] Benjamini Y Yekutieli D False discovery rate-adjusted multiple confidence intervals for selected parameters JASA 2005 100 71 93 10.1198/016214504000001907

[B32] Benjamini Y Hochberg Y Controlling the false discovery rate: a practical and powerful approach to multiple testing JRSS B 1995 57 289 300

[B33] Harrison DA Lertsithichai P Brady AR Carpenter JR Rowan K Winter excess mortality in intensive care in the UK: an analysis of outcome adjusted for patient casemix and unit workload Int Care Med 2004 30 1900 7 10.1007/s00134-004-2390-6 15300367

[B34] Twisk J de Boer M de Vente W Heymans M Multiple imputation of missing values was not necessary before performing a longitudinal mixed-model analysis J Clin Epi 2013 66 1022 28 10.1016/j.jclinepi.2013.03.017 23790725

[B35] ANZICS CORE ANZICS Centre for Outcome and Resource Evaluation 2011 Outlier management policy at: [ http://www.anzics.com.au/core/core-management-governance] 10.1186/1471-2288-14-53 PMC4021168 24755369

