# Supplementary material for: Identifying unusual performance in Australian and New Zealand intensive care units from 2000 to 2010
Source: BMC Med Res Methodol. 2014 Apr 22;14:53. doi: 10.1186/1471-2288-14-53 (PMC4021168; doi:10.1186/1471-2288-14-53)
Supplement: Additional file 1 — Variance of the log-SMR. The file SE-logSMR.pdf outlines the calculations required to obtain an expression for the approximate variance of the log-SMR for ICU i in year t. The approximate standard error of the log-SMR is obtained by taking the square root of the variance and is used in the time-plots in Figure 6. [file 1471-2288-14-53-S1.pdf]

Approximate variance of the log-SMR

The random variable representing the mortality outcomes for patients is assumed to be $Y_{itj} = 1$ if patient j in year t in ICU i died in hospital, and 0 otherwise, $j = 1, \ldots, n_{it}$, $t = 1, \ldots, n_i$, $i = 1, \ldots, 144$.

The individual patient mortality outcomes are then modelled by
$Y_{itj}|\{X_{itj}, Z_{itj}, W_{itj}, U_{tj0}, U_i\} \sim \text{Bernoulli}(P_{itj})$, where

$$P_{itj} = \frac{\exp\left(\beta^T X_{itj} + U_{it}^T Z_{itj} + U_i^T W_{itj}\right)}{1 + \exp\left(\beta^T X_{itj} + U_{it}^T Z_{tji} + U_i^T W_{itj}\right)}, \quad U_{it} \sim N_{p_1+1}(0, \Sigma_2), \quad U_i \sim N_{p_2+1}(0, \Sigma_3).$$

The p-vector X_{itj} contains the (fixed-effects) explanatory variables, the (p_1+1)-vector Z_{itj} contains the data for the level two (ICU-year level) random effects, and W_{itj} is the (p_2+1)-vector containing the data for the level three (ICU-level) random effects. The U_{it} are the ICU-year level random effects and the U_i are the ICU-level random effects.

The log-SMR for ICU i in year t is given by:

$$S_{it} = \log\left(\sum_{j=1}^{n_{it}} Y_{itj}\right) - \log\left(\sum_{j=1}^{n_{it}} P_{itj}\right),$$

$t = 1, \ldots, n_i$, $i = 1, \ldots, 144$, which is estimated by

$$\hat{S}_{it} = \log\left(\sum_{i=1}^{n_{it}} Y_{itj}\right) - \log\left(\sum_{i=1}^{n_{it}} \hat{P}_{itj}\right) = \log\left(O_{jt}\right) - \log\left(\hat{E}_{jt}\right),$$

where

$$\hat{P}_{itj} = \frac{\exp\left(\hat{\beta}^T X_{itj} + \hat{U}_{it}^T Z_{itj} + \hat{U}_i^T W_{itj}\right)}{1 + \exp\left(\hat{\beta}^T X_{itj} + \hat{U}_{it}^T Z_{itj} + \hat{U}_i^T W_{itj}\right)};$$

$\hat{U}_{it}$ is the empirical Bayesian modal prediction of U_{it}, [3]. To simplify the following development, $U_{it}^T Z_{itj} + U_i^T W_{itj}$ is written as $U_{it}^{*T} Z_{itj}^*$.

The variance of $\hat{S}_{it}$ may be written

$$\begin{aligned}\mathrm{var}\left(\hat{S}_{it}\right) &= \mathrm{var}\left\{\log\left(\frac{O_{it}}{\hat{E}_{it}}\right)\right\} = \mathrm{var}\left\{\log(O_{it}) - \log(\hat{E}_{it})\right\} \\ &= \mathrm{var}\left\{\log(O_{it})\right\} + \mathrm{var}\left\{\log(\hat{E}_{it})\right\} - 2\mathrm{cov}\left\{\log(O_{it}), \log(\hat{E}_{it})\right\}.\end{aligned}$$

If each ICU in each year has a small number of patient admissions relative to the total number of patient admissions, the covariance term can be assumed to be zero. Then, following [2], delta-method arguments give

$$\mathrm{var}\left(\hat{S}_{it}\right) \approx \frac{\mathrm{var}(O_{it})}{O_{it}^2} + \frac{\mathrm{var}(\hat{E}_{it})}{\hat{E}_{it}^2}.$$

Expressions for $\mathrm{var}(O_{it})$ and $\mathrm{var}(\hat{E}_{it})$ are then required. Now,

$$\mathrm{var}(O_{it}) = \mathrm{var}\left(\sum_{j=1}^{n_{it}} Y_{itj}\right) = \sum_{j=1}^{n_{it}} \mathrm{var}\left(Y_{itj}\right) + 2\sum_{j<k} \mathrm{cov}(Y_{itj}, Y_{itk}).$$

To find estimators for these variances and covariances, we use Goldstein's linearisation [1, p128]:

$$\begin{aligned}Y_{itj} &= \left(\beta^T X_{itj} + U_{it}^{*T} Z_{itj}^*\right) \frac{\exp\left(\beta^T X_{itj}\right)}{\left[1+\exp\left(\beta^T X_{itj}\right)\right]^2} + e_{itj}\sqrt{\tilde{P}_{itj}(1-\tilde{P}_{itj})}, \\ \text{where} \quad \tilde{P}_{itj} &= \frac{\exp\left(\beta^T X_{itj}\right)}{1+\exp\left(\beta^T X_{itj}\right)}, \quad \mathrm{var}(e_{itj}) = 1.\end{aligned}$$

An expression for the variance of the outcome for patient j in year t, ICU i is then

$$\begin{aligned}\mathrm{var}\left(Y_{itj}\right) =& \tilde{P}_{itj}^2(1-\tilde{P}_{itj})^2 \mathrm{var}\left(\beta^T X_{itj} + U_{it}^{*T} Z_{itj}^*\right) + \tilde{P}_{itj}(1-\tilde{P}_{itj}) \\ =& \tilde{P}_{itj}^2(1-\tilde{P}_{itj})^2 Z_{itj}^{*T} \mathrm{var}\left(U_{it}^*\right) Z_{itj}^* + \tilde{P}_{itj}(1-\tilde{P}_{itj}), \\ =& \tilde{P}_{itj}^2(1-\tilde{P}_{itj})^2 \left\{Z_{itj}^T \mathrm{var}\left(U_{it}\right) Z_{itj} + W_{itj}^T \mathrm{var}\left(U_i\right) W_{itj}\right\} + \tilde{P}_{itj}(1-\tilde{P}_{itj}),\end{aligned}$$

which may be estimated by

$$\widehat{\mathrm{var}}\left(Y_{itj}\right) = \hat{\tilde{P}}_{itj}^2(1-\hat{\tilde{P}}_{itj})^2 \left[Z_{itj}^T \hat{\Sigma}_2 Z_{itj} + W_{itj}^T \hat{\Sigma}_3 W_{itj}\right] + \hat{\tilde{P}}_{itj}(1-\hat{\tilde{P}}_{itj}), \quad \hat{\tilde{P}}_{itj} = \frac{\exp\left(\hat{\beta}^T X_{itj}\right)}{1+\exp\left(\hat{\beta}^T X_{itj}\right)}.$$

Again using Goldstein's linearisation to obtain estimates of the covariances of the outcomes

for two patients in ICU i in year t,

$$\begin{aligned}
\operatorname{cov}(Y_{itj}, Y_{itk}) &= E\left[\left(Y_{itj} - E[Y_{itj}]\right)\left(Y_{itk} - E[Y_{itk}]\right)\right] \\
&= E\left[\left\{ Z_{itj}^{*T} U_{it}^{*} \frac{\exp\left(X_{itj}^{T}\beta\right)}{\left[1+\exp\left(X_{itj}^{T}\beta\right)\right]^{2}} + e_{itj}\sqrt{\tilde{P}_{itj}(1-\tilde{P}_{itj})} \right\}\right. \\
&\quad \left. \times \left\{ Z_{itk}^{*T} U_{it}^{*} \frac{\exp\left(X_{itk}^{T}\beta\right)}{\left[1+\exp\left(X_{itk}^{T}\beta\right)\right]^{2}} + e_{itk}\sqrt{\tilde{P}_{itk}(1-\tilde{P}_{itk})} \right\}\right] \\
&= E\left[\frac{\tilde{P}_{itj}}{1+\exp\left(X_{itj}^{T}\beta\right)} \frac{\tilde{P}_{itk}}{1+\exp\left(X_{itk}^{T}\beta\right)} Z_{itj}^{*T} U_{it}^{*} Z_{itk}^{*T} U_{it}^{*} \right] \\
&= \tilde{P}_{itj}(1-\tilde{P}_{itj})\tilde{P}_{itk}(1-\tilde{P}_{itk}) Z_{itj}^{*T} E\left[U_{it}^{*} U_{it}^{*T}\right] Z_{itk},
\end{aligned}$$

which can be estimated by

$$\widehat{\operatorname{cov}}(Y_{itj}, Y_{itk}) = \hat{\hat{P}}_{itj}(1-\hat{\hat{P}}_{itj})\hat{\hat{P}}_{itk}(1-\hat{\hat{P}}_{itk}) \left(Z_{itj}^{T}\hat{\Sigma}_2 Z_{itk} + W_{itj}^{T}\hat{\Sigma}_3 W_{itk} \right).$$

An expression for $\operatorname{var}(\hat{E}_{it})$ is now required:

$$\operatorname{var}(\hat{E}_{it}) = \operatorname{var}\left(\sum_{j=1}^{n_{it}} \hat{P}_{itj}\right) = \sum_{j=1}^{n_{it}} \operatorname{var}(\hat{P}_{itj}) + 2\sum_{j<k} \operatorname{cov}(\hat{P}_{itj}, \hat{P}_{itk}).$$

To simplify the expression, let $X_{itj}^{*}\hat{\beta}_{it}^{*} = (X_{itj}, Z_{itj}, W_{itj})(\hat{\beta}, \hat{U}_{it}, \hat{U}_{i})^{T}$ and $\hat{P}_{it} = (\hat{P}_{itj}, \ldots, \hat{P}_{itn_{it}})^{T}$. Then $\operatorname{var}(\hat{E}_{it})$ is the sum of the elements of the covariance matrix of $\hat{P}_{it}$. Once again following [2] and using a delta-method argument,

$$\operatorname{var}(\hat{P}_{it}) = \left(\frac{\partial P_{it}}{\partial \hat{\beta}_{it}^{*}}\right)^{T} \operatorname{var}(\hat{\beta}_{it}^{*}) \left(\frac{\partial P_{it}}{\partial \hat{\beta}_{it}^{*}}\right), \quad \frac{\partial \hat{P}_{itj}}{\partial (\hat{\beta}_{it}^{*})_{k}} = x_{k}^{*}\hat{P}_{itj}(1-\hat{P}_{itj}),$$

where x_k^* is the kth element of X_{itj}^* and $(\hat{\beta}_{it}^*)_k$ is the kth element of $\hat{\beta}_{it}^*$. The approximate variance of the estimated log-SMR of ICU i in year t can then be expressed as

$$\begin{aligned}
\operatorname{var}\left(\hat{S}_{it}\right) \approx & \frac{1}{O_{it}^{2}} \sum_{j=1}^{n_{it}} \sum_{k=1}^{n_{it}} \hat{\hat{P}}_{itj}(1-\hat{\hat{P}}_{itj})\hat{\hat{P}}_{itk}(1-\hat{\hat{P}}_{itk}) \left(Z_{itj}^{T}\hat{\Sigma}_2 Z_{itk} + W_{itj}^{T}\hat{\Sigma}_3 W_{itk} \right) + \frac{1}{O_{it}^{2}} \sum_{j=1}^{n_{it}} \hat{\hat{P}}_{itj}(1-\hat{\hat{P}}_{itj}) \\
& + \frac{1}{\hat{E}_{it}^{2}} \sum_{j=1}^{n_{it}} \sum_{k=1}^{n_{it}} \hat{P}_{itj}(1-\hat{P}_{itj})\hat{P}_{itk}(1-\hat{P}_{itk}) X_{itj}^{T*} \operatorname{var}(\hat{\beta}^{*}) X_{itk}^{*}.
\end{aligned}$$

The approximate standard error is obtained by taking the square root of the variance.

These results can be generalized to m-level hierarchical models, although fitting models with additional levels in the hierarchy may prove to be computationally very intensive and time consuming for many applications.

References

[1] H. Goldstein. *Multilevel Statistical Models*. 4th Ed. Wiley: Chichester, West Sussex, 2010.

[2] D. W. Hosmer and S. Lemeshow. Confidence interval estimates of an index of quality performance based on logistic regression models. *Statist Med*, **14**:2161–72, 1995.

[3] S. Rabe-Hesketh and A. Skrondal. *Multilevel and longitudinal modeling using Stata*, Vol. II: Categorical responses, counts and survival. Stata Press: College Station, TX, 2012.
