# Supplementary material for: Identifying unusual performance in Australian and New Zealand intensive care units from 2000 to 2010
Source: BMC Med Res Methodol. 2014 Apr 22;14:53. doi: 10.1186/1471-2288-14-53 (PMC4021168; doi:10.1186/1471-2288-14-53)
Supplement: Additional file 2 — Table of Stage 1 and Stage 2 model parameter estimates. Stages 1 and 2.pdf presents all fixed effects parameter estimates (as log odds) from the three-level hierarchical logistic regression models fitted in Stages 1 and 2 of the analysis. The interpretation for the categorical variables is the increase in log-odds of the in-hospital mortality relative to the baseline level (given in brackets). The p-value in each case gives the two-sided probability of observing the estimate, or one that is more extreme, under the null hypothesis that the log odds ratio equals zero. Corresponding estimated 95% confidence intervals are also given. [file 1471-2288-14-53-S2.pdf]

Table 1: Stage 1 and Stage 2 model parameter estimates. For categorical variables, the interpretation is the increase in log-odds of in-hospital mortality relative to the baseline level given in brackets. For example, being a medical cardiovascular patient versus a medical respiratory patient, decreases the log odds of in-hospital mortality by 0.3016.

Variable	Stage 1 model				Stage 2 model			
	Log odds	p-value	95% CI		Log odds	p-value	95% CI	
**Patient-level variables**:								
APACHE III score	1.9933	< 0.0001	1.9035	2.0830	1.9706	< 0.0001	1.8730	2.0681
APACHE III^2	-0.0233	0.1282	-0.0534	0.0067	-0.0425	0.0128	-0.0760	-0.0091
APACHE III^3	-0.0734	< 0.0001	-0.0922	-0.0545	-0.0561	< 0.0001	-0.0768	-0.0354
APACHE III^4	0.0160	< 0.0001	0.0125	0.0194	0.0127	< 0.0001	0.0089	0.0164
Age< 50	0.0212	< 0.0001	0.0186	0.0238	0.0208	< 0.0001	0.0179	0.0236
$50 \leq$ Age < 80	0.0209	< 0.0001	0.0194	0.0224	0.0206	< 0.0001	0.0190	0.0223
$80 \leq$ Age < 85	0.0232	< 0.0001	0.0219	0.0245	0.0231	< 0.0001	0.0217	0.0246
$85 \leq$ Age < 90	0.0202	< 0.0001	0.0188	0.0216	0.0202	< 0.0001	0.0187	0.0217
$90 \leq$ Age < 95	0.0228	< 0.0001	0.0212	0.0245	0.0225	< 0.0001	0.0207	0.0243
Age≥ 95	0.0210	< 0.0001	0.0181	0.0239	0.0216	< 0.0001	0.0185	0.0247
APACHE III score $\times$ Age< 50	-0.0069	< 0.0001	-0.0089	-0.0049	-0.0061	< 0.0001	-0.0083	-0.0039
APACHE III score $\times$ $50 \leq$ Age < 80	-0.0073	< 0.0001	-0.0085	-0.0061	-0.0069	< 0.0001	-0.0081	-0.0056
APACHE III score $\times$ $80 \leq$ Age < 85	-0.0070	< 0.0001	-0.0080	-0.0060	-0.0068	< 0.0001	-0.0079	-0.0056
APACHE III score $\times$ $85 \leq$ Age < 90	-0.0061	< 0.0001	-0.0072	-0.0051	-0.0059	< 0.0001	-0.0071	-0.0047
APACHE III score $\times$ $90 \leq$ Age < 95	-0.0060	< 0.0001	-0.0074	-0.0047	-0.0055	< 0.0001	-0.0069	-0.0040
APACHE III score $\times$Age≥ 95	-0.0068	< 0.0001	-0.0091	-0.0044	-0.0067	< 0.0001	-0.0092	-0.0042

Continued on next page

Table 1 – continued from previous page

Variable	Stage 1 model				Stage 2 model			
Sex (male)	-0.1045	< 0.0001	-0.1357	-0.0734	-0.1041	< 0.0001	-0.1380	-0.0701
Diagnostic Category (Respiratory(med))								
Cardiovascular (medical)	-0.3016	< 0.0001	-0.3592	-0.2440	-0.2858	< 0.0001	-0.3492	-0.2225
Liver/Gastrointestinal (medical)	-0.5493	< 0.0001	-0.6217	-0.4768	-0.5585	< 0.0001	-0.6368	-0.4803
Central Nervous System (medical)	0.0497	0.1827	-0.0234	0.1228	0.0579	0.1563	-0.0222	0.1380
Sepsis (medical)	-0.6381	< 0.0001	-0.7044	-0.5718	-0.6440	< 0.0001	-0.7157	-0.5723
Trauma (medical)	-0.9150	< 0.0001	-1.0413	-0.7888	-1.0017	< 0.0001	-1.1415	-0.8619
Metabolic hormonal (medical)	-1.7847	< 0.0001	-1.9072	-1.6621	-1.7287	< 0.0001	-1.8587	-1.5987
Haematologic (medical)	0.0859	0.2760	-0.0686	0.2404	0.0592	0.4920	-0.1097	0.2282
Renal/Genitourinary (medical)	-0.9099	< 0.0001	-1.0273	-0.7925	-0.9149	< 0.0001	-1.0418	-0.7879
Other medical disorders	-0.8728	< 0.0001	-1.0551	-0.6905	-0.8134	< 0.0001	-1.0052	-0.6217
Cardiovascular (elective)	-1.9923	< 0.0001	-2.1001	-1.8844	-1.9652	< 0.0001	-2.0840	-1.8465
Thoracic (elective)	-1.1982	< 0.0001	-1.3150	-1.0813	-1.1639	< 0.0001	-1.2962	-1.0316
Gastrointestinal (elective)	-1.2040	< 0.0001	-1.2765	-1.1315	-1.1839	< 0.0001	-1.2620	-1.1059
Central Nervous System (elective)	-1.3930	< 0.0001	-1.5364	-1.2497	-1.2152	< 0.0001	-1.3849	-1.0455
Traumatic/Orthopaedic (elective)	-1.6940	< 0.0001	-1.8232	-1.5648	-1.6043	< 0.0001	-1.7442	-1.4643
Renal/Genitourinary (elective)	-2.0995	< 0.0001	-2.3002	-1.8989	-2.0931	< 0.0001	-2.3095	-1.8766
Gynaecological/Oncological (elective)	-2.2025	< 0.0001	-2.5497	-1.8553	-2.1965	< 0.0001	-2.5806	-1.8125
Cardiovascular (emergency)	-0.9064	< 0.0001	-1.0390	-0.7738	-0.9172	< 0.0001	-1.0611	-0.7733
Thoracic (emergency)	-0.7469	< 0.0001	-0.9361	-0.5578	-0.7248	< 0.0001	-0.9341	-0.5155
Gastrointestinal (emergency)	-0.9234	< 0.0001	-0.9965	-0.8502	-0.9332	< 0.0001	-1.0122	-0.8542
Central Nervous System(emergency)	-0.2144	0.0050	-0.3642	-0.0647	-0.1399	0.0980	-0.3057	0.0258
Traumatic/Orthopaedic (emergency)	-1.1522	< 0.0001	-1.2728	-1.0316	-1.1779	< 0.0001	-1.3090	-1.0469
Renal/Genitourinary (emergency)	-1.8425	< 0.0001	-2.1313	-1.5537	-1.8615	< 0.0001	-2.1795	-1.5435

Continued on next page

Table 1 – continued from previous page

Variable	Stage 1 model				Stage 2 model			
Gynaecological/Oncological (emergency)	-2.1652	< 0.0001	-2.6868	-1.6436	-2.2759	< 0.0001	-2.8559	-1.6959
Ventilation	0.0815	0.0040	0.0260	0.1370	0.1149	0.0002	0.0546	0.1753
ICU source (no transfer)	-0.1099	< 0.0001	-0.1555	-0.0643	-0.1372	< 0.0001	-0.1881	-0.0864
Ventilation × APACHE III	-0.2925	< 0.0001	-0.3230	-0.2621	-0.3179	< 0.0001	-0.3512	-0.2847
Ventilation × sex	0.0703	0.0008	0.0292	0.1114	0.0696	0.0023	0.0249	0.1142
Ventilation × Diagnostic category								
Ventilation × Cardiovascular (medical)	0.2153	< 0.0001	0.1390	0.2916	0.2258	< 0.0001	0.1427	0.3089
Ventilation × Liver/Gastrointestinal (medical)	0.1924	0.0004	0.0858	0.2989	0.2275	0.0001	0.1121	0.3429
Ventilation × Central Nervous System (medical)	0.4944	< 0.0001	0.4052	0.5836	0.4948	< 0.0001	0.3979	0.5918
Ventilation × Sepsis (medical)	0.4047	< 0.0001	0.3196	0.4899	0.4103	< 0.0001	0.3180	0.5026
Ventilation × Trauma (medical)	0.4114	< 0.0001	0.2627	0.5600	0.4831	< 0.0001	0.3182	0.6464
Ventilation × Metabolic hormonal (medical)	-0.1259	0.1091	-0.2799	0.0281	-0.1288	0.1279	-0.2947	0.0370
Ventilation × Haematologic (medical)	0.9210	< 0.0001	0.6813	1.1606	0.9851	< 0.0001	0.7279	1.2423
Ventilation × Renal/Genitourinary (medical)	0.3838	< 0.0001	0.1990	0.5686	0.4264	< 0.0001	0.2283	0.6244
Ventilation × Other medical disorders	-0.0734	0.6364	-0.3779	0.2310	-0.1293	0.4341	-0.4535	0.1948
Ventilation × Cardiovascular (elective)	-0.0365	0.5853	-0.1678	0.0947	-0.1037	0.1589	-0.2480	0.0406
Ventilation × Thoracic (elective)	0.1593	0.1220	-0.0426	0.3612	0.1808	0.1150	-0.0440	0.4057
Ventilation × Gastrointestinal (elective)	0.3163	< 0.0001	0.2033	0.4294	0.3105	< 0.0001	0.1890	0.4320
Ventilation × Central Nervous System (elective)	0.9896	< 0.0001	0.7648	1.2144	0.8778	< 0.0001	0.6205	1.1351
Ventilation × Traumatic/Orthopaedic (elective)	0.4052	0.0031	0.1370	0.6734	0.4088	0.0049	0.1241	0.6936
Ventilation × Renal/Genitourinary (elective)	0.0148	0.9425	-0.3887	0.4184	0.0844	0.7004	-0.3454	0.5142
Ventilation × Gynaecological/Oncological (elective)	0.1864	0.5986	-0.5076	0.8804	0.0393	0.9233	-0.7620	0.8406
Ventilation × Cardiovascular (emergency)	0.0154	0.8478	-0.1414	0.1721	0.0189	0.8268	-0.1501	0.1878
Ventilation × Thoracic (emergency)	0.0448	0.7121	-0.1931	0.2827	0.0835	0.5319	-0.1782	0.3451

Continued on next page

Table 1 – continued from previous page

Variable	Stage 1 model				Stage 2 model			
Ventilation × Gastrointestinal (emergency)	0.2768	< 0.0001	0.1861	0.3675	0.2945	< 0.0001	0.1968	0.3923
Ventilation × Central Nervous System(emergency)	0.6596	< 0.0001	0.4888	0.8303	0.6091	< 0.0001	0.4219	0.7963
Ventilation × Traumatic/Orthopaedic (emergency)	0.5018	< 0.0001	0.3596	0.6441	0.5369	< 0.0001	0.3832	0.6905
Ventilation × Renal/Genitourinary (emergency)	0.5003	0.0069	0.1372	0.8634	0.5150	0.0095	0.1257	0.9043
Ventilation × Gynaecological/Oncological (emergency)	0.3341	0.3192	-0.3233	0.9914	0.4854	0.1781	-0.2211	1.1919
Diagnostic category × APACHE III score								
Cardiovascular (medical) × APACHE III score	0.2655	< 0.0001	0.2255	0.3056	0.2544	< 0.0001	0.2109	0.2980
Liver/Gastrointestinal (medical) × APACHE III score	0.3039	< 0.0001	0.2453	0.3624	0.3010	< 0.0001	0.2374	0.3647
Central Nervous System (medical) × APACHE III score	0.0703	0.0035	0.0231	0.1174	0.0685	0.0092	0.0170	0.1200
Sepsis (medical) × APACHE III score	0.1163	< 0.0001	0.0696	0.1630	0.1158	< 0.0001	0.0651	0.1665
Trauma (medical) × APACHE III score	0.4915	< 0.0001	0.4199	0.5631	0.5021	< 0.0001	0.4238	0.5804
Metabolic hormonal (medical) × APACHE III score	0.2677	< 0.0001	0.1927	0.3428	0.2323	< 0.0001	0.1519	0.3128
Haematologic (medical) × APACHE III score	-0.1443	0.0151	-0.2607	-0.0279	-0.1668	0.0101	-0.2937	-0.0398
Renal/Genitourinary (medical) × APACHE III score	-0.0437	0.3432	-0.1341	0.0467	-0.0547	0.2675	-0.1515	0.0420
Other medical disorders × APACHE III score	0.3454	< 0.0001	0.1942	0.4966	0.3098	0.0002	0.1493	0.4702
Cardiovascular (elective)× APACHE III score	0.5479	< 0.0001	0.4705	0.6254	0.6309	< 0.0001	0.5443	0.7175
Thoracic (elective) × APACHE III score	0.1633	0.0193	0.0265	0.3001	0.1568	0.0464	0.0025	0.3111
Gastrointestinal (elective) × APACHE III score	0.0546	0.1258	-0.0153	0.1245	0.0559	0.1446	-0.0192	0.1311
Central Nervous System (elective) × APACHE III score	0.0298	0.6928	-0.1182	0.1779	0.0366	0.6743	-0.1340	0.2071
Traumatic/Orthopaedic (elective) × APACHE III score	0.3520	< 0.0001	0.2003	0.5037	0.3128	< 0.0001	0.1482	0.4774
Renal/Genitourinary (elective) × APACHE III score	0.2164	0.0628	-0.0116	0.4443	0.1753	0.1659	-0.0727	0.4232
Gynaecological/Oncological (elective) × APACHE III score	0.6027	0.0049	0.1824	1.0230	0.6830	0.0089	0.1710	1.1949
Cardiovascular (emergency) × APACHE III score	0.3121	< 0.0001	0.2368	0.3873	0.3296	< 0.0001	0.2480	0.4112
Thoracic (emergency) × APACHE III score	0.1402	0.0324	0.0118	0.2687	0.1255	0.0804	-0.0152	0.2663

Continued on next page

Table 1 – continued from previous page

Variable	Stage 1 model				Stage 2 model			
Gastrointestinal (emergency) × APACHE III score	0.1452	< 0.0001	0.0973	0.1931	0.1426	< 0.0001	0.0907	0.1944
Central Nervous System(emergency) × APACHE III score	0.0986	0.0187	0.0164	0.1808	0.0859	0.0566	-0.0024	0.1742
Traumatic/Orthopaedic (emergency) × APACHE III score	0.3046	< 0.0001	0.2290	0.3802	0.3067	< 0.0001	0.2250	0.3884
Renal/Genitourinary (emergency) × APACHE III score	0.2858	0.0042	0.0900	0.4816	0.3395	0.0021	0.1228	0.5561
Gynaecological/Oncological (emergency) × APACHE III score	0.3595	0.0073	0.0968	0.6222	0.3417	0.0143	0.0683	0.6151
ICU source × APACHE III score	-0.0900	< 0.0001	-0.1220	-0.0581	-0.0709	0.0001	-0.1064	-0.0354
Weekly period cosine	0.0287	0.0264	0.0034	0.0541	0.0276	0.0396	0.0013	0.0538
Weekly period sine	0.0017	0.9000	-0.0246	0.0279	-0.0039	0.7773	-0.0310	0.0232
Yearly period cosine	-0.0434	0.0010	-0.0692	-0.0176	-0.0389	0.0043	-0.0656	-0.0122
Yearly period sine	0.0163	0.2172	-0.0096	0.0421	0.0181	0.1836	-0.0086	0.0449
**ICU-year-level variables**:								
Annual Volume	0.0000	0.4170	-0.0001	0.0001	0.0001	0.0968	-0.0000	0.0002
Year	-0.0569	< 0.0001	-0.0777	-0.0361	-0.0625	< 0.0001	-0.0845	-0.0406
Year squared	-0.0000	0.9572	-0.0018	0.0017	0.0004	0.7047	-0.0015	0.0022
**ICU-level variables**:								
Hospital level (Tertiary)								
Rural	-0.0350	0.6356	-0.1797	0.1098	0.0326	0.5520	-0.0748	0.1400
Metropolitan	-0.0715	0.2991	-0.2065	0.0635	-0.0155	0.7698	-0.1191	0.0882
Private	-0.3417	< 0.0001	-0.4790	-0.2044	-0.2629	< 0.0001	-0.3760	-0.1499
Locality (NSW)								
Northern Territory	-0.1791	0.3844	-0.5826	0.2244	-0.2320	0.1185	-0.5192	0.0588
Australian Capital Territory	-0.3431	0.0562	-0.6954	0.0091	-0.2093	0.1505	-0.4946	0.0760
South Australia	0.0859	0.4832	-0.1543	0.3262	-0.1324	0.4131	-0.4496	0.1847
Victoria	-0.0502	0.5034	-0.1973	0.0968	-0.1783	0.0039	-0.2994	-0.0573

Continued on next page

Table 1 – continued from previous page

Variable	Stage 1 model				Stage 2 model			
Western Australia	1.0957	0.0079	0.2873	1.9041	1.1788	0.0018	0.4385	1.9191
New Zealand	0.0938	0.4740	-0.1630	0.3505	0.0705	0.5119	-0.1402	0.2811
Queensland	0.0375	0.6294	-0.1147	0.1896	-0.0217	0.7402	-0.1502	0.1067
Tasmania	0.0610	0.7272	-0.2816	0.4035	0.0169	0.8943	-0.2328	0.2666
Locality × year								
Northern Territory × year	0.0267	0.1298	-0.0079	0.0614	0.0273	0.1222	-0.0073	0.0620
Australian Capital Territory × year	0.0189	0.2456	-0.0130	0.0508	0.0216	0.1923	-0.0109	0.0541
South Australia × year	-0.0140	0.2065	-0.0358	0.0077	-0.0101	0.6302	-0.0512	0.0310
Victoria × year	0.0089	0.1986	-0.0047	0.0225	0.0147	0.0397	0.0007	0.0286
Western Australia × year	-0.1166	0.0207	-0.2154	-0.0178	-0.1399	0.0030	-0.2324	-0.0474
New Zealand × year	0.0085	0.5126	-0.0169	0.0338	0.0078	0.5429	-0.0173	0.0328
Queensland × year	-0.0067	0.3580	-0.0208	0.0075	-0.0124	0.1077	-0.0275	0.0027
Tasmania × year	-0.0025	0.8682	-0.0325	0.0274	-0.0022	0.8846	-0.0322	0.0278
Locality × Weekly cosine term								
Northern Territory	0.0130	0.8168	-0.0967	0.1226	0.0146	0.7947	-0.0952	0.1244
Australian Capital Territory	0.0194	0.6707	-0.0719	0.1107	0.0289	0.5441	-0.0645	0.1223
South Australia	-0.0057	0.8253	-0.0562	0.0449	0.0113	0.7824	-0.0686	0.0912
Victoria	-0.0275	0.1439	-0.0643	0.0094	-0.0259	0.1875	-0.0645	0.0126
Western Australia	0.0072	0.9198	-0.1333	0.1477	0.0068	0.9240	-0.1337	0.1474
New Zealand	-0.0376	0.2218	-0.0980	0.0227	-0.0353	0.2545	-0.0961	0.0254
Queensland	-0.0262	0.2064	-0.0668	0.0144	-0.0328	0.1442	-0.0769	0.0112
Tasmania	-0.0901	0.0560	-0.1825	0.0023	-0.0895	0.0582	-0.1822	0.0031
Locality × Weekly sine term								
Northern Territory	-0.0232	0.6838	-0.1346	0.0883	-0.0172	0.7627	-0.1289	0.0945

Continued on next page

Table 1 – continued from previous page

Variable	Stage 1 model				Stage 2 model			
Australian Capital Territory	0.0168	0.7293	-0.0784	0.1120	0.0033	0.9464	-0.0935	0.1001
South Australia	0.0262	0.3216	-0.0256	0.0781	0.0555	0.1838	-0.0263	0.1373
Victoria	0.0030	0.8783	-0.0351	0.0411	0.0098	0.6310	-0.0300	0.0496
Western Australia	0.1499	0.0419	0.0055	0.2943	0.1566	0.0337	0.0121	0.3010
New Zealand	-0.0104	0.7436	-0.0730	0.0521	-0.0038	0.9060	-0.0667	0.0591
Queensland	0.0479	0.0268	0.0055	0.0902	0.0564	0.0157	0.0107	0.1022
Tasmania	-0.0314	0.5160	-0.1263	0.0634	-0.0244	0.6156	-0.1195	0.0707
Locality × Yearly cosine term								
Northern Territory	0.0416	0.4594	-0.0687	0.1519	0.0372	0.5088	-0.0732	0.1477
Australian Capital Territory	-0.0819	0.0821	-0.1743	0.0104	-0.0847	0.0775	-0.1787	0.0093
South Australia	0.0374	0.1524	-0.0138	0.0897	0.0552	0.1805	-0.0256	0.1361
Victoria	0.0204	0.2849	-0.0170	0.0579	0.0225	0.2604	-0.0167	0.0616
Western Australia	0.0316	0.6617	-0.1100	0.1732	0.0248	0.7316	-0.1168	0.1664
New Zealand	-0.0164	0.6021	-0.0779	0.0452	-0.0206	0.5146	-0.0825	0.0413
Queensland	0.0426	0.0287	0.0048	0.0876	0.0385	0.0919	-0.0063	0.0834
Tasmania	0.0744	0.1227	-0.0201	0.1689	0.0707	0.1437	-0.0241	0.1655
Locality × Yearly sine term								
Northern Territory	-0.0164	0.7717	-0.1273	0.0945	-0.0184	0.7458	-0.1295	0.0927
Australian Capital Territory	-0.0892	0.0628	-0.1831	0.0048	-0.0964	0.0491	-0.1923	-0.0004
South Australia	-0.0016	0.9503	-0.0532	0.0499	0.0205	0.6211	-0.0608	0.1018
Victoria	0.0124	0.5188	-0.0252	0.0499	0.0084	0.6742	-0.0308	0.0477
Western Australia	-0.0872	0.2305	-0.2296	0.0553	-0.0899	0.2163	-0.2325	0.0526
New Zealand	0.0415	0.1862	-0.0200	0.1030	0.0393	0.2133	-0.0226	0.1012
Queensland	0.0141	0.5068	-0.0275	0.0556	0.0256	0.2652	-0.0194	0.0706

Continued on next page

Table 1 – continued from previous page

Variable	Stage 1 model				Stage 2 model			
Tasmania	0.0371	0.4315	-0.0554	0.1297	0.0352	0.4576	-0.0576	0.1280
Intercept	-2.9442	< 0.0001	-3.1120	-2.7764	-2.9029	< 0.0001	-3.0580	-2.7478
