# Supplementary figures and images for: Identifying unusual performance in Australian and New Zealand intensive care units from 2000 to 2010
Source: BMC Med Res Methodol. 2014 Apr 22;14:53. doi: 10.1186/1471-2288-14-53 (PMC4021168; doi:10.1186/1471-2288-14-53)

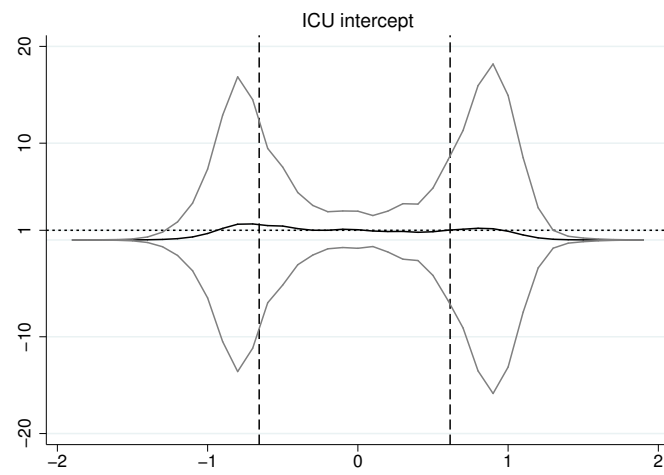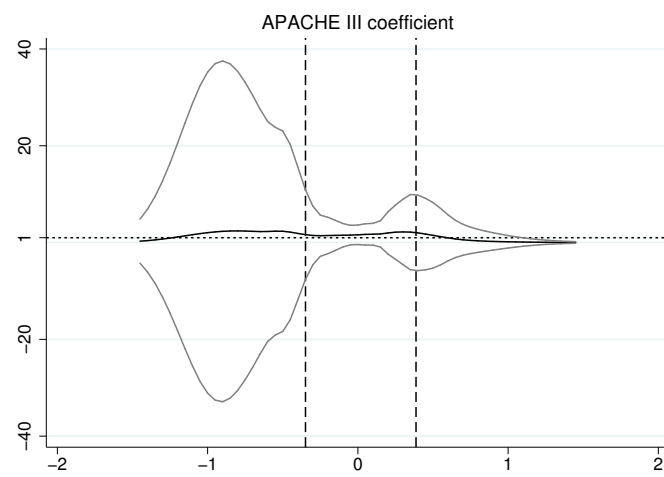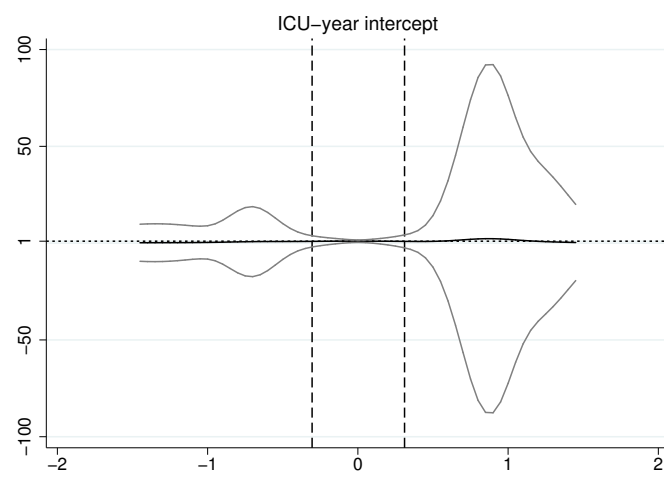

Supplement: Additional file 3 — Estimated gradient function plots. GradFct.pdf displays the estimated gradient functions for the random effects from the Stage 1 hierarchical model, with point wise confidence intervals in grey, [30]. These plots assess the assumptions of normality made in the model. Normality is assumed to be reasonable if the estimated function (solid line in each case) is close to one (horizontal dotted line) within the limits of the observed data as represented by the vertical dashed lines in each sub-plot. If the estimated gradient function is significantly different from one, the assumption of normality is not valid. The subplots shown in order are for the level-three intercepts, the level-three APACHE III random slopes, and the ICU-year intercepts. As would be expected for such a large dataset as the ANZICS APD, the normality assumptions are satisfied here. Note that a simplifying assumption has been made for the plots presented, in particular, that the random intercepts and random slopes are independent at level-three (ICU-level) of the model. [file 1471-2288-14-53-S3.pdf]
